# Evaluation of nutritional adequacy in adult patients with Crohn’s disease: a cross-sectional study

**DOI:** 10.1007/s00394-020-02198-0

**Published:** 2020-02-18

**Authors:** Iolanda Cioffi, Nicola Imperatore, Olivia Di Vincenzo, Maria Carmen Pagano, Lidia Santarpia, Lucienne Pellegrini, Anna Testa, Maurizio Marra, Franco Contaldo, Fabiana Castiglione, Fabrizio Pasanisi

**Affiliations:** 1 Internal Medicine and Clinical Nutrition Unit, Department of Clinical Medicine and Surgery, Federico II University Hospital, via S. Pansini 5, 80131 Naples, Italy; 2 Gastroenterology Unit, Department of Clinical Medicine and Surgery, Federico II University Hospital, via Pansini 5, 80131 Naples, Italy

**Keywords:** Crohn’s disease, Nutritional assessment, Inflammatory bowel disease, Micronutrient, Dietary intake

## Abstract

**Purpose:**

Inadequate oral intake may play an important role in the onset of malnutrition in patients with Crohn’s disease (CD). The aims of this cross-sectional study were: (1) to compare dietary intake in clinically active and quiescent CD patients, and (2) to assess patients’ nutritional adequacy relative to the dietary reference values (DRVs) for the Italian population using LARN (Livelli di Assunzione di Riferimento di Nutrienti ed energia per la popolazione italiana).

**Methods:**

Patients aged between 18 and 65 years with a diagnosis of CD were recruited**.** All participants underwent anthropometry and were instructed to fill in a 3-day food record. Disease activity was clinically defined using the Crohn’s disease activity index (CDAI).

**Results:**

Overall, 117 patients, 71 males and 46 females, with a mean age of 39.6 ± 13.8 years and a mean body weight of 65.4 ± 11.8 kg, were ultimately included. Our findings showed that the amount of nutrients was similar between patients with active and quiescent disease. The mean intake of macronutrients was adequate, except for fiber, while dietary micronutrients were insufficient. Median intakes of sodium, phosphorus, and fluorine met LARN recommendations in both sexes, and the DRVs were accomplished by many patients (53/117; 104/117 and 98/117, respectively). Interestingly, dietary amounts of iron and zinc were barely acceptable in males but not in females. However, a few of the patients (< 15) met the LARN for potassium, calcium, and magnesium, regardless of sex and CDAI. With respect to vitamins, no relevant difference was found between the active and quiescent groups, and none of them met recommended values in both sexes.

**Conclusions:**

This study showed that the assessment of dietary intake can be crucial for optimizing dietary intervention with focused nutrition counseling, to improve nutritional status in CD patients.

## Introduction

Crohn's disease (CD) is an idiopathic chronic inflammatory disease able to affect any segment of the gastrointestinal tract, eliciting persistent transmural inflammation with consequent structural bowel damage and intestinal complications such as strictures, fistulae, and abscesses, which often require surgery [1, 2]. Generally, CD patients are at risk of nutritional deficiencies both during periods of disease exacerbation (due to reduced oral intake, malabsorption, and nutrient losses through the gut) and remission [3–5]. Specifically, poor and/or inadequate oral intake may play an important role in the onset of malnutrition due to many factors such as voluntary food restrictions, increased satiety, reduced sensation of pleasure related to eating, changes in mood, and even medical advice [6, 7].

Currently, reduced macronutrient intakes are less common in these patients, whereas deficits in micronutrients, such as iron, copper, selenium, magnesium, zinc, vitamins, and antioxidants (vitamins C, E, beta carotene, glutathione, taurine) can occur frequently [8–16], even in apparently well-nourished patients [13]. Some food group restrictions, such as dairy products and/or fruit and vegetables, are pursued by patients to control their symptoms [10, 17]. Additionally, appetite sensation could dramatically change, especially with active disease, in which patients experience less hunger and more satiety [18]. As such, knowledge regarding reduced and/or inadequate food intake in CD patients can contribute to the early identification of nutritional deficiencies. Basically, quantifying nutrient intake in the context of the overall nutrition status assists in determining if patients are meeting recommended levels of nutrient intake and to what extent subjects should require specific supplementation [19]. To date, there are limited published data describing the dietary patterns as well as macro- and micro-nutrient intakes in patients with CD [20, 21] and no data are available in Italian individuals with CD.

Therefore, the objectives of the present study were: (1) to compare energy and nutrient intake, collected by 3-day food records, in clinically active and quiescent CD patients, classified according to the Crohn’s disease activity index (CDAI) and (2) to assess their nutritional adequacy compared to the dietary reference values (DRVs) for the Italian population using LARN (Livelli di Assunzione di Riferimento di Nutrienti ed energia per la popolazione italiana) [22], according to sex and specific-age ranges.

## Subjects and methods

We evaluated differences in dietary intake and adequacy by studying food records prospectively collected from a cohort of CD patients participating in the REECD study (Resting Energy Expenditure evaluation in subjects with Crohn's Disease). Consecutive patients aged between 18 and 65 years with a diagnosis of CD were recruited at the Department of Clinical Medicine of Federico II University Hospital in Naples between July 2016 and March 2018. The exclusion criteria were the following: use of corticosteroids in the last 3 months, history of acute or chronic liver or kidney disease, current enteral and parenteral nutrition, intestinal fistulae, ileostomy or colostomy, presence of extensive small bowel resections (residual small bowel < 2 m), pregnancy or lactation, unstable body weight in the last month, inability or unwillingness to give informed consent.

The Crohn’s disease activity index (CDAI) was adopted to classify patients in the active and remission phases (≥ 150 and < 150, respectively). Additionally, clinical data such as disease diagnosis, location, drug treatment, disease duration etc. were collected. All participants provided written informed consent prior to enrolment. All procedures were conducted in accordance with the Helsinki Declaration of 1975 as revised in 1983 and were approved by the Ethics Committee of Federico II University (Protocol’s number: 102/16). This study was registered at clinicaltrials.gov as NCT03054935.

### Energy intake and nutrient assessment

All patients were instructed by a registered dietitian, face-to-face or by phone, to fill in a food diary for 3 nonconsecutive days (2 weekdays and 1 weekend day) before coming to the hospital. Specifically, participants were taught how to estimate food portions using household measurements such as bowls, cups, spoons, and plates. A dedicated dietitian reviewed the completed 3-day food diary upon return for clarification of portions, missing or unclear data, and food preparation methods. All diaries were calculated using the WINFOOD database (3.4 version; Medimatica, Teramo, Italy).

### Comparison to the dietary reference values for the Italian population

DRVs are an umbrella term that include the population reference intakes (PRIs), the average requirements (ARs), adequate intakes (AIs) and reference intake (RIs) ranges for macronutrients [23]. They can be used to identify nutrient intakes that are relevant for planning dietary treatments and research purposes in individuals as well as in population groups [22, 23]. LARN (Livelli di Assunzione di Riferimento di Nutrienti ed energia per la popolazione italiana) are the last version of the Italian DRVs released in 2014 by the Italian Society of Human Nutrition [22], in line with the EFSA (European Food Safety Authority) technical report [23]. Thus, the PRI is the level of nutrient intake that is adequate for the majority of people in a population group. The AR is the intake level that is adequate to meet the physiological needs of half of the individuals in a population. When there is insufficient scientific evidence to estimate the AR or PRI, AI is established by estimating the intake of an apparently healthy population group that is assumed to have adequate intake. Finally, RI is the intake range for macronutrients, expressed as % of the energy intake; and corresponds to ranges that are adequate for maintaining health.

As suggested by technical reports [22, 23], to evaluate the adequacy of macro- and micronutrient intake, data obtained from 3-day food records were compared with AR or RI values provided by LARN. However, when the AR values were not available, we referred to AI values for comparison. Specifically, AI was used for sodium, potassium, manganese, iodine, molybdenum, fluorine, vitamin E, vitamin K and biotin levels.

### Statistical analysis

Data were expressed either as the means and standard deviation (SD) or as medians and ranges, depending on the distribution of the data. Differences between groups were assessed with unpaired *t* tests and analysis of variance for parametric variables; while the Mann–Whitney *U* test or Kruskal–Wallis was applied for nonparametric data. The chi-squared test was used for differences between categorical variables. The number and percentage of patients whose reported intakes were higher or lower than the AR, RI or AI values were calculated for each micronutrient. Statistical analyses were performed using SPSS version 22.0 (IBM Corporation, Inc. Chicago, IL, USA) and the significance level was set at the *p* < 0.05 level.

## Results

Overall, 148 CD patients were recruited for participation, but 31 were ruled out for the following reasons: 8 subjects did not fulfil the inclusion criteria, 11 did not return their 3-day food records, and 12 supplemented with protein and/or micronutrients during the study. Therefore, a total of 117 patients, 71 males and 46 females, were finally included in this analysis.

The demographic and clinical characteristics of the patients are shown in Table 1. Overall, CD patients had a mean age of 39.6 ± 13.8 years and an average body weight of 65.4 ± 11.8 kg. Based on the CDAI, 53 patients were clinically active (score ≥ 150), while 64 were in the quiescent phase (score < 150). The disease duration was approximately 8 years in both sexes. Based on the Montreal classification, CD was mainly diagnosed between 17 and 40 years (66%), located in the ileum—colon (53%) and characterized by a stricturing phenotype (50%). Regarding drug therapies, patients were treated with biologic agents (41%), immunosuppressives (19%), mesalazine (16%), and almost 30% of them were out of therapy at the time of the study visit, due to the screening phase before starting biologic therapy.

**Table 1:** Clinical characteristics of CD patients

	Total	Men	Women
*N*, (%)	117 (100)	71 (61)	46 (39)
Age, years [SD]	39.6 [13.8]	38.6 [13.8]	41.1 [13.9]
BMI, kg/m^2^ [SD]	23.2 [3.4]	23.7 [2.26]	22.5 [3.69]
Mean disease duration, y [range]	8.23 [0.5–30]	8.80 [1–30]	7.36 [0.5–23]
Clinical activity, *n* (%)			
CDAI < 150	64 (55)	43 (61)	21 (46)
CDAI > 150	53 (45)	28 (39)	25 (54)
Montreal age at diagnosis, *n* (%)			
A1: < 16 y	21 (18)	14 (20)	7 (15)
A2: 17–40 y	77 (66)	47 (66)	30 (65)
A3: > 40 y	19 (16)	10 (14)	9 (20)
Montreal disease location, *n* (%)			
L1: Ileum	41 (35)	24 (34)	17 (37)
L2: Colon	11 (9)	10 (14)	1 (2)
L3: Ileum and colon	62 (53)	35 (49)	27 (59)
L4: Upper GI tract	3 (3)	2 (3)	1 (2)
Montreal disease behaviour, *n* (%)			
B1: Inflammatory	36 (31)	27 (38)	9 (20)
B2: Stricturing	59 (50)	35 (49)	24 (52)
B3: Penetrating	22 (19)	9 (13)	13 (28)
Perianal disease, *n* (%)	23 (20)	13 (18)	10 (22)
Medications, *n* (%)			
None	34 (29)	21 (30)	13 (28)
5-ASA	19 (16)	12 (17)	7 (17)
IMMs	16 (14)	8 (11)	8 (15)
Biologics	48 (41)	30 (42)	18 (39)

*ASA* amino salicylic acid, *CDAI* Crohn disease activity index, *IMMs* immunosuppressives

No differences were found for age and BMI between males and females (Table 1), whereas body weight differed (males = 70.5 ± 10.5 kg vs. females = 57.6 ± 9.37 kg; *p* = 0.03). Moreover, disease activity assessed by the CDAI was higher in females compared to males (males: 124 ± 74 vs. females: 160 ± 83; *p* = 0.04).

Data on self-reported energy and dietary intakes are presented separated by sex and disease activity (active and quiescent groups).

### Self-reported energy intake (EI) and macronutrients

Among CD patients, daily self-reported EI did not differ between the active and quiescent phases for either males or females, as reported in Tables 2 and 3, respectively. However, it was lower than the EI recommended by LARN by almost 500 kcal for both sexes.

**Table 2:** Daily energy and macronutrient intake as well as their adequacy in male CD patients, according to disease activity

	LARN		All (*n* = 71)	EI^b^	Active (*n* = 28)	EI^b^	Quiescent (*n* = 43)	EI^b^
	RI	AR	Mean ± SD	%	Mean ± SD	%	Mean ± SD	%
Energy intake (EI), kcal/d		2350^c^	1863 ± 345		1915 ± 451		1829 ± 260	
Protein, g		50^d^	79.0 ± 23.6	17%	80.1 ± 29.1	17%	78.3 ± 19.6	17%
Protein g/kg body weight		0.71	0.98 ± 0.36		0.98 ± 0.41		0.99 ± 0.34	
Fat, g	25–35% EI		63.9 ± 18.8	32%	64.4 ± 21.2	31%	63.6 ± 17.4	30%
SFA, g	< 10% EI		17.3 ± 7.53	8%	17.3 ± 9.14	8%	17.3 ± 6.89	8%
MUFA, g	–		26.4 ± 8.41	14%	23.9 ± 8.68*	13%	28.1 ± 7.91	12%*
PUFA, g	5–10% EI		6.58 ± 2.56	3.2%	6.41 ± 3.05	3%	6.70 ± 2.21	3.0%
n-6 PUFA, g	4–8% EI		3.36 ± 1.74	1.7%	3.18 ± 2.02	1.6%	3.47 ± 1.54	1.5%
n-3 PUFA, g	0.5–2% EI		0.39 ± 0.28	0.2%	0.42 ± 0.40	0.2%	0.38 ± 0.16	0.2%
Cholesterol, mg	< 300		219 ± 102		204 ± 110		229 ± 97	
Carbohydrate, g	45–60% EI		255 ± 55.6	51%	268 ± 70.8	52%	247 ± 41.8	53%
Starch, g	–		155 ± 45.8		161 ± 50.6		152 ± 42.7	
Oligosaccharides, g	< 15% EI		56.8 ± 29.7	11%	59.8 ± 31.9	11%	54.8 ± 28.4	11%
Fiber/1000 kcal, g	12.6–16.7		7.23 ± 2.50		7.25 ± 2.34		7.22 ± 2.71	
Total fibers, g^a^	25		13.5 ± 5.22		14.1 ± 5.52		13.1 ± 5.03	
Soluble, g	–		2.94 ± 1.46		2.91 ± 1.39		2.98 ± 1.58	
Insoluble, g	–		5.13 ± 3.11		5.20 ± 3.34		5.01 ± 2.78	

*LARN* Livelli di Assunzione di Riferimento di Nutrienti ed energia per la popolazione italiana, *RI* Reference intake ranges for macronutrients, *AR* average requirements, *SFA *saturated fatty acid, *MUFA* monounsaturated fatty acid, *PUFA* polyunsaturated fatty acid, *EI* energy intake

*p < 0.05

^a^Value suggested for disease prevention

^b^Macronutrients expressed as percentage of total EI

^c^Energy needs associated with the lowest level of physical activity [14]

^d^Based on a body weight of 70 kg

**Table 3:** Daily energy and macronutrient intakes as well as their adequacy in female CD patients, according to disease activity

	LARN		All (*n* = 46)	EI^b^	Active (*n* = 25)	EI^b^	Quiescent (*n* = 21)	EI^b^
	RI	AR	Mean ± SD	%	Mean ± SD	%	Mean ± SD	%
Energy intake (EI), kcal/d		1900^c^	1445 ± 222		1440 ± 224		1452 ± 224	
Protein, g		43^d^	61.1 ± 15.0	17%	60.7 ± 16.4	17%	61.7 ± 13.5	17%
Protein, g/kg body weight		0.71	1.00 ± 0.32		1.01 ± 0.32		1.00 ± 0.32	
Fat, g	25–35% EI		52.5 ± 12.9	33%	51.5 ± 13.2	33%	53.6 ± 12.7	32%
SFA, g	< 10% EI		13.8 ± 5.20	8%	14.7 ± 5.83	9%	12.7 ± 4.23	9%
MUFA, g	–		22.5 ± 6.13	15%	21.1 ± 6.04*	14%	24.2 ± 5.94	13%*
PUFA, g	5–10% EI		5.30 ± 2.24	3.7%	4.73 ± 1.53*	3.3%	5.98 ± 2.65	2.9%*
n-6 PUFA, g	4–8% EI		2.97 ± 1.56	2%	2.69 ± 0.97	1.9%	3.30 ± 2.04	1.7%
n-3 PUFA, g	0.5–2% EI		0.36 ± 0.29	0.2%	0.30 ± 0.12	0.2%	0.42 ± 0.39	0.2%
Cholesterol, mg	< 300		173 ± 99		192 ± 115		152 ± 73	
Carbohydrate, g	45–60% EI		193 ± 39.0	49%	193 ± 32.5	50%	192 ± 46.4	51%
Starch, g	–		111 ± 29.2		110 ± 22.7		112 ± 36.0	
Oligosaccharides, g	< 15% EI		42.3 ± 20.0	10%	45.7 ± 20.0	11%	38.3 ± 19.7	12%
Fiber/1000 kcal, g	12.6–16.7		6.21 ± 2.14		6.02 ± 2.04		6.54 ± 2.26	
Total fibers, g^a^	25		8.93 ± 3.14		8.56 ± 2.94		9.38 ± 3.39	
Soluble, g	–		2.08 ± 0.99		1.81 ± 0.79*		2.39 ± 1.12	
Insoluble, g	–		3.48 ± 2.13		2.94 ± 1.69*		4.13 ± 2.46	

*LARN* Livelli di Assunzione di Riferimento di Nutrienti ed energia per la popolazione italiana, *RI* Reference intake ranges for macronutrients, *AR* average requirements, *SFA *saturated fatty acid, *MUFA* monounsaturated fatty acid, *PUFA* polyunsaturated fatty acid, *EI* energy intake

*p < 0.05

^a^Value suggested for disease prevention

^b^Macronutrients expressed as percentage of total EI

^c^Energy needs considering the lowest level of physical activity [14]

^d^Based on a body weight of 60 kg

In the same tables, we showed macronutrient intakes, according to the CDAI. Overall, total protein, protein per kilogram of body weight as well as fat and carbohydrate intakes were within the range proposed by LARN for males and females; while dietary fiber and polyunsaturated fatty acids (PUFAs), including both n-6 and n-3 PUFAs, were lower than the recommended values. In male CD patients, based on the CDAI, no difference was observed for macronutrient intake, except for monounsaturated fatty acid (MUFA), which was lower for those in the active group than those in the quiescent group (active: 23.9 ± 8.68 g vs. quiescent: 28.1 ± 7.91 g; *p* = 0.04). Among female CD patients, we found lower intakes for MUFA, total PUFA and soluble and insoluble dietary fibers in the active group than in the quiescent group (Table 3). Overall, the mean value for dietary intake of cholesterol was below the cutoff range in both sexes (< 300 mg), regardless of CDAI; however, almost 15% of patients had high intakes.

When macronutrients were assessed as a percentage of total energy intake, we found a similar distribution between males and females (Tables 2 and 3) as well as between CDAI groups (data not shown)**.** Generally, we noted a slight increase in total fat intake (> 30%) in all patients, with greater consumption of MUFA (> 12%) compared to PUFAs, while saturated fatty acid (SFA) was less than 10% of total EI, as recommended by LARN [21].

### Minerals and trace elements

Both minerals and trace elements for male and female CD patients and the number of subjects meeting the LARN recommendations are shown in Tables 4 and 5, respectively. Median intakes of sodium, phosphorus, iron, copper, and fluorine met LARN recommendations and were achieved by most patients (43/71; 66/71; 44/71; 44/71 and 66/71, respectively). Zinc intake was higher in 29/71 patients, while potassium, calcium, magnesium, iodine, selenium and manganese intakes were considerably lower than the recommended level. Indeed, a small number of subjects (< 15) met the LARN values and none of them met the molybdenum dietary recommendation (0/71). Based on the CDAI, we did not find any differences in median mineral intake, but the number of patients who met the LARN recommendations slightly varied according to disease activity. Specifically, we observed that none of the patients in the quiescent group achieved recommendations for potassium (0/43) and iodine (0/43).

**Table 4:** Minerals and trace elements intake in male CD patients, according to disease activity

	LARN		All (*n* = 71)		Active (*n* = 28)		Quiescent (*n* = 43)	
	AR	AI	Median (min–max)	No. meeting LARN	Median	No. meeting LARN	Median	No. meeting LARN
Sodium (mg)**		1500	1631 (318–4629)	43 (61%)	1589	16 (57%)	1655	27 (63%)
Potassium (mg)		3900	1799 (867–4198)	2 (3%)	1840	2 (7%)	1799	0
Calcium (mg)**	800		351 (64–1194)	4 (6%)	357	2 (7%)	346	2 (5%)
Phosphorus (mg)	580		921 (312–2193)	66 (93%)	852	25 (89%)	921	41 (95%)
Magnesium (mg)	170		128 (39–270)	14 (20%)	133	6 (21%)	123	8 (19%)
Iron (mg)**	7		7.85 (2–26)	44 (62%)	8.01	18 (64%)	7.82	26 (61%)
Zinc (mg)	10		8.55 (3–21)	29 (41%)	8.04	10 (36%)	8.71	19 (44%)
Iodine (mg)		150	42.1 (1.2–226)	3 (4%)	43.8	3 (11%)	42.1	0
Copper (mg)	0.7		0.84 (0.2–7.0)	44 (62%)	0.78	17 (61%)	0.86	27 (63%)
Selenium (µg)	45		19.2 (3.4–103)	9 (13%)	13.0	3 (11%)	22.6	6 (14%)
Fluorine (mg)		3.5	24.2 (0–206)	66 (76%)	27.0	25 (68%)	24.1	41 (79%)
Manganese (mg)		2.7	0.93 (0–28.5)	6 (9%)	1.03	2 (7%)	0.93	4 (9%)
Molybdenum (µg)		65	1.43 (0–17.7)	0	1.12	0	1.46	0

Data are expressed both as number and percentage of subjects meeting LARN (Livelli di Assunzione di Riferimento di Nutrienti ed energia per la popolazione italiana)

*AR* average requirement, *AI* adequate intake

*p < 0.05

**Values considered for age-range

**Table 5:** Minerals and trace elements intake in female CD patients, according to disease activity

	LARN		All (*n* = 46)		Active (*n* = 25)		Quiescent (*n* = 21)	
	AR	AI	Median (min–max)	No. meeting LARN	Median	No. meeting LARN	Median	No. meeting LARN
Sodium (mg)**		1500	1045 (20–2506)	9 (20%)	1106	7 (28%)	935	2 (10%)
Potassium (mg)		3900	1344 (636–2422)	0	1398	0	1295	0
Calcium (mg)**	800		338 (37–1101)	3 (7%)	380	2 (8%)	331	1 (5%)
Phosphorus (mg)	580		743 (230–1307)	38 (83%)	766	21 (84%)	742	17 (81%)
Magnesium (mg)	170		97.4 (37–210)	1 (2%)	92.8	0	101	1 (5%)
Iron (mg)**	10		6.47 (2–11)	4 (9%)	6.41	3 (12%)	6.53	1 (5%)
Zinc (mg)	8		7.53 (3–14)	21 (46%)	7.68	11 (44%)	7.14	10 (48%)
Iodine (mg)		150	33.4 (0–75)	0	29.8	0	44.1	0
Copper (mg)	0.7		0.67 (0.1–1.6)	21 (46%)	0.59*	8 (32%)	0.74	13 (62%)
Selenium (µg)	45		19.5 (1.8–52.8)	3 (7%)	20.5	1 (4%)	18.5	2 (10%)
Fluorine (mg)		3	28.2 (0–107)	32 (70%)	29.2	15 (60%)	28.2	17 (81%)
Manganese (mg)		2.3	0.78 (0–30.1)	8 (17%)	0.73	5 (20%)	0.81	3 (14%)
Molybdenum (µg)		65	2.36 (0–21)	0	2.22	0	2.58	0

Data are expressed both as number and percentage of subjects meeting LARN (Livelli di Assunzione di Riferimento di Nutrienti ed energia per la popolazione italiana)

*AR* average requirement, *AI* adequate intake

*p < 0.05

**Values considered for age-range

In female CD patients, intakes of phosphorus, zinc, copper, and fluorine were good for most patients, whereas potassium, calcium, magnesium, iron, iodine, selenium, and molybdenum intakes were low compared to the recommended values. Specifically, none of them met the LARN recommendations for potassium, iodine or molybdenum intake (0/46). Based on the CDAI, we found a lower intake of copper in the active group than in the quiescent group (active = 0.59 mg vs. quiescent = 0.74 mg; *p* = 0.02).

**Fig. 1:**
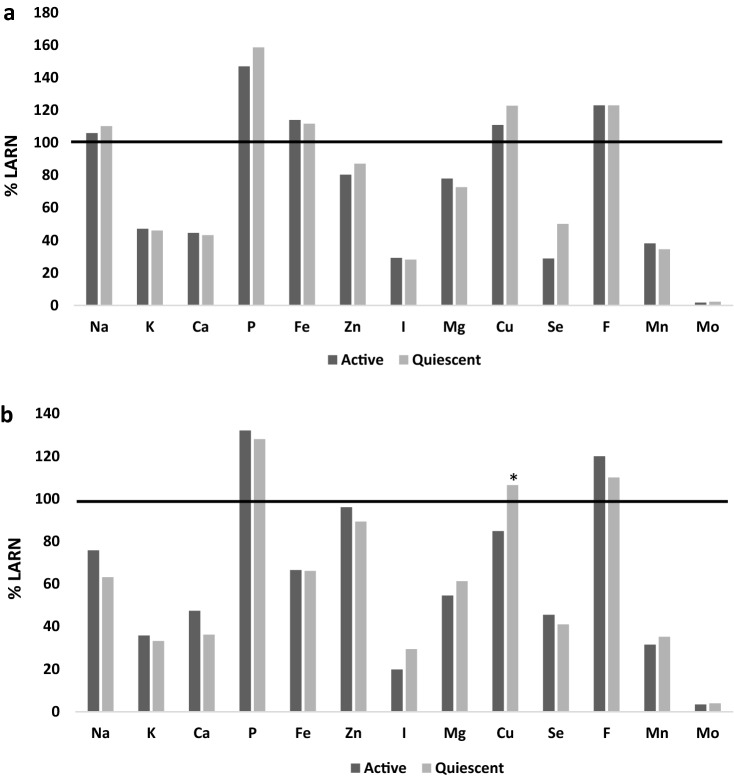
Minerals and trace elements. The percentage values of mineral and trace element intake were compared to the LARN values (average requirements or adequate intake) in both male (*n* = 71) and female (*n* = 46) CD patients, according to disease activity, in panels **a** and **b**, respectively. Data are shown as the median intake. *p < 0.05

When intakes were expressed as a percentage of the LARN recommendations, the data clearly showed that only phosphorous, copper, and fluorine met the LARN recommended values in both CDAI groups for males and females, while sodium and iron were achieved in male CD patients only (Fig. 1). As reported in Fig. 1, copper intake was significantly different between percentage values in female CD patients (*p* = 0.02).

### Vitamins

In Table 6, we presented vitamin intakes for both males (a) and females (b) as well as the number of subjects meeting the LARN values. The median intakes of all vitamins were low and unrelated to CDAI. None of the male participants met the LARN recommendations for biotin intake, while a small number of patients (< 7) met the vitamin D, vitamin E, vitamin K, vitamin B5 and folate recommendations. No significant difference was observed between the CDAI groups.

**Table 6:** Vitamin intake in male CD patients, according to disease activity

	LARN		All (*n* = 71)		Active (*n* = 28)		Quiescent (*n* = 43)	
	AR	AI	Median (min–max)	No. meeting LARN	Median	No. meeting LARN	Median	No. meeting LARN
Vitamin A (µg)	500		328 (15.2–4896)	14 (20%)	328	5 (19%)	334	9 (21%)
Vitamin D (µg)**	10		1.35 (0–11.9)	1 (1%)	1.54	0	1.31	1 (2%)
Vitamin E (mg)		13	7.78 (2–16.9)	6 (9%)	7.28	2 (7%)	8.10	4 (9%)
Vitamin K (µg)**		140	6.25 (0–173)	2 (3%)	5.17	0	8.98	2 (5%)
Thiamin (mg)	1.0		0.71 (0.4–1.8)	22 (31%)	0.67	11 (39%)	0.72	11 (26%)
Riboflavin (mg)	1.3		0.97 (0.3–13.3)	24 (34%)	0.86	9 (32%)	1.03	15 (35%)
Niacin (mg)	14		12.3 (3.3–33.3)	30 (42%)	12.9	12 (43%)	12.3	18 (42%)
Vitamin B5 (mg)		5	1.16 (0.2–3.10)	2 (3%)	1.14	1 (4%)	1.21	1 (2%)
Vitamin B6 (mg)**	1.1		1.25 (0–4-3.0)	46 (65%)	1.23	16 (57%)	1.29	30 (70%)
Biotin (µg)		30	4.71 (0–28.3)	0	4.71	0	4.82	0
Folate (µg)	320		140 (34–376)	4 (6%)	152	2 (7%)	140	2 (5%)
Vitamin B12 (µg)	2.0		0.96 (0–116)	21 (30%)	0.96	9 (32%)	0.78	12 (28%)
Vitamin C (mg)	75		48.5 (0–277)	26 (37%)	39.5	11 (39%)	51.4	15 (35%)

Data are expressed both as number and percentage of subjects meeting LARN (Livelli di Assunzione di Riferimento di Nutrienti ed energia per la popolazione italiana)

*AR* average requirement, *AI* adequate intake

*p < 0.05

**Values considered for age-range

Similar results were observed in females, where the intakes of vitamin E (active = 6.01 mg vs. quiescent = 7.54 mg; *p* = 0.03) and niacin (active = 9.46 mg vs. quiescent = 11.9 mg; *p* = 0.04) were significantly lower in the active group than in the quiescent group. Again, none of patients (0/46) met the LARN recommendations for vitamin K, vitamin B5, biotin or folate, and the number of patients meeting LARN was low for the remaining vitamins, especially in the active group, where none of them achieved the LARN values for vitamin D and vitamin E (Table 7).

**Table 7:** Vitamin intake in female CD patients, according to disease activity

	LARN		All (*n* = 46)		Active (*n* = 25)		Quiescent (*n* = 21)	
	AR	AI	Median (min–max)	No. meeting LARN	Median	No. meeting LARN	Median	No. meeting LARN
Vitamin A (µg)	400		268 (2–4560)	11 (24%)	310	7 (28%)	251	4 (19%)
Vitamin D (µg) **	10		1.55 (0–15.4)	2 (%)	1.44	0	1.73	2 (10%)
Vitamin E (mg)		12	6.58 (1.6–13.3)	1 (2%)	6.01*	0	7.54	1(5%)
Vitamin K (µg) **		140	6.83 (0–116)	0	7.12	0	6.54	0
Thiamin (mg)	0.9		0.63 (0.2–1.4)	8 (17%)	0.65	5 (20%)	0.61	3 (14%)
Riboflavin (mg)	1.1		0.86 (0.2–6.5)	12 (26%)	0.99	8 (32%)	0.81	4 (19%)
Niacin (mg)	14		10.7 (3.8–22.9)	14 (30%)	9.46*	5 (20%)	11.9	9 (43%)
Vitamin B5 (mg)		5	0.98 (0.3–3.3)	0	0.92*	0	1.18	0
Vitamin B6 (mg)**	1.1		1.04 (0.5–3.4)	17 (37%)	1.03	7 (28%)	1.05	10 (48%)
Biotin (µg)		30	4.85 (0–25)	0	4.49	0	5.50	0
Folate (µg)	320		121 (34–260)	0	115	0	134	0
Vitamin B12 (µg)	2.0		1.04 (0–19.4)	10 (22%)	1.08	6 (24%)	0.81	4 (19%)
Vitamin C (mg)	60		31.4 (0–250)	12 (26%)	30.9	8 (32%)	31.9	4 (19%)

Data are expressed both as number and percentage of subjects meeting LARN (Livelli di Assunzione di Riferimento di Nutrienti ed energia per la popolazione italiana)

*AR* average requirement, *AI* adequate intake

*p < 0.05

**Values considered for age-range

**Fig. 2:**
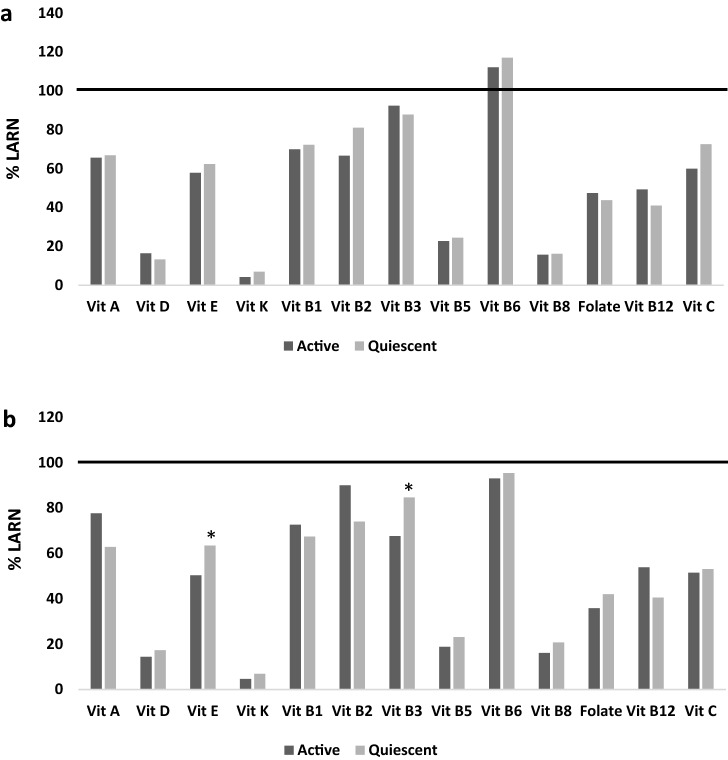
Vitamins. The percentage values of vitamin intake were compared to the LARN values (average requirements or adequate intake) in both male (*n* = 71) and female (*n* = 46) CD patients, according to disease activity, in panels **a** and **b**, respectively. Data are shown as the median intake. *p < 0.05

By expressing vitamin intake as a percentage of the LARN recommendations, as shown in Fig. 2, we found that none of the suggested values for vitamin were met by patients, except for vitamin B6 in males. On the other hand, in females, both vitamin E and niacin were significantly higher for the quiescent compared to the active group (*p* = 0.03 and *p* = 0.04, respectively).

## Discussion

The aims of the present study were first to compare macro- and micronutrient intakes using 3-day food records in active and quiescent CD patients, and second to assess nutritional adequacy by comparing their intakes with the LARN recommendations. Our findings showed that both energy and nutrient intakes were similar between patients with active and quiescent disease. However, most patients failed to meet the LARN recommendations for micronutrients, unrelated to sex and disease activity, suggesting that symptoms of active CD might not be the major factor limiting nutritional intake.

As previously described [6], low and/or inadequate nutrient intake can contribute considerably to the onset of malnutrition in CD patients; therefore, their investigation would be useful for planning effective dietary strategies and supplementation, when indicated. To date, however, only a few studies assessed dietary intake in these patients, agreeing with altered micronutrient intakes when compared to established recommended values [19, 21] as well as to a national survey [20, 24]. In the present study, self-reported EI resulted in a lower calorie intake by approximately 500 kcal compared to the EI suggested by LARN [22], who considered the daily energy needs estimated by a predictive equation, not measured; therefore, EI was lower than that theoretically required. Nevertheless, the macronutrient distribution met LARN values for protein, fat, and carbohydrate; and a high intake of MUFA (> 12%), which differed between active and quiescent patients, and low intake of SFA (< 10%), according to the recommendations, were reported. Likewise, previous Canadian studies showed adequate intakes of protein and carbohydrate in CD patients, but a different fat distribution, with increasing SFA [18–20] and decreasing MUFA intakes [19, 20], likely due to different dietary habits. Regarding PUFA intake, including the essential fatty acids (n-6 and n-3), we found that they were below the cutoff values proposed by LARN, and that the ratio of n-6/n-3 was in favor of n-6 PUFAs (9:1). Previously, Taylor et al. [20] described a lower percentage of both n-6 and n-3 PUFAs in CD patients compared to the Canadian Community Health Survey [25].

An imbalance in the ratio of n-6/n-3 PUFAs, in favor of n-6, is typically observed in the Western diet. Specifically, n-6 PUFAs have pro-inflammatory properties, since they are precursors of arachidonic acid, which is metabolized into thromboxane A2, leukotriene B4, and prostaglandins [26], and have been shown to modulate the intestinal inflammatory response [27]. Therefore, an increase in the consumption of animal protein (meat), fast foods, refined grains and certain margarines, typical of a westernized diet, has been associated with high dietary intake of n-6 PUFAs [28]. In contrast, dietary n-3 PUFAs such as eicosapentaenoic acid (EPA) and docosahexaenoic acid (DHA), which are mainly concentrated in fish oils, salmon, tuna, nuts, flaxseeds etc., have been shown to have anti-inflammatory effects in several chronic inflammatory disorders [29], and are involved in the regulation of immunological and inflammatory responses [28, 29]. To date, experimental evidences has shown that n-3 PUFAs could have a protective role on CD risk and that high intakes might reduce flares [29]; however, data are still controversial and conflicting on the effect of n-3 and n-6 PUFAs in CD patients [30]. The results from 3-day food records showed that our patients preferred animal proteins, derived mostly from meat rather than fish, compared to other sources of protein; potentially explaining the pro-inflammatory n-6/n-3 ratio. Additionally, we found that females in the active group ate less plant-based protein compared to those in the quiescent group (active: 15.5 ± 4.63 g vs. quiescent: 18.8 ± 5.99 g; *p* = 0.03).

Regarding the intake of dietary fiber, expressed as both the total and grams per 1000 kcal/day, we observed reduced values (7.2 g/1000 kcal) compared to the LARN recommendation (RI =  ~ 15 g/1000 kcal), even in patients in clinical remission. Previous studies showed an average daily intake of fiber/1000 kcal that ranged from 6.13 [31] to 11.1 g [20], without showing any differences between active and quiescent groups [21], in agreement with our results.

Generally, CD patients had a low fiber intake because many physicians prescribed a low-residue diet to reduce diarrhea or avoid abdominal symptoms due to strictures and bacterial overgrowth. In fact, the consumption of vegetables, fruit peels, nuts, and seeds provides insoluble fibers that are metabolically fermented in the colon and can accelerate the movement of bolus through the intestine [21]; this is harmful in patients with severe flares and in those with stricturing type and obstructive symptoms [32, 33]. Unfortunately, some patients extend the low-residue diet long after its prescription has ended, avoiding any form of legumes, vegetables and fruit, since they are afraid of eating food that can be linked with exacerbation of their symptoms. Accordingly, we did not find any differences in fiber intake between patients who showed a stricturing type (B2: fiber = 6.7 ± 1.7 g/1000 kcal) and the others (B1 + B3: fiber = 7.7 ± 3.0 g/1000 kcal, based on Montreal classification). Moreover, a low fiber intake can also be explained by the fact that patients are not routinely followed by a nutritional team during the disease course, and they base their diet on personal experiences and/or information drawn from the media [31].

Therefore, low diet diversity along with self-imposed and prolonged food group exclusion can be responsible for several nutritional deficits [20, 21, 31]. Our results showed that only sodium, phosphorus, iron, copper and fluorine intake met the LARN recommendations, while potassium, calcium, magnesium, zinc, selenium, iodine, manganese and molybdenum were inadequate. Similarly, the majority of vitamin intakes were insufficient to achieve the LARN recommendations, regardless of disease activity. These results can be explained by the reduced amounts of plant-based foods, which represented the main dietary sources of minerals and vitamins. When we looked at the number of subjects meeting the LARN values, we found that only a small number of patients (< 15) achieved the LARN recommended values for micronutrients, with the highest number of subjects reported for sodium (52/117), phosphorus (104/117), fluorine (98/117) and vitamin B6 (62/117).

These findings partially agreed with previous data [19–21] showing that CD patients are frequently exposed to insufficient micronutrient intakes. Therefore, it is useful to identify any micronutrient shortfalls to treat them. For instance, supplementation of vitamin C and vitamin E has previously been shown to result in a reduction of oxidative stress in CD patients [34]. However, adequate intake of zinc could be associated with improvement of clinical outcomes; since zinc plays a pivotal role in wound repair, tissue regeneration, and the immune system [35].

Finally, we briefly compared the dietary data of our group of CD patients with data collected by the third Italian National Food Consumption Survey, INRAN-SCAI 2005-06 Italian Survey [36], in adult Italian people (18–65 years). We found that macronutrients, especially total fat and SFA intakes, were higher compared both to those reported by CD patients as well as those suggested by LARN, while carbohydrates were lower. Regarding dietary fiber, the daily intake was higher than that observed for CD patients, without meeting the LARN recommendations. The mean daily intakes of phosphorus, magnesium, iron and zinc were adequate in both sexes, apart from iron in females; while calcium and potassium were insufficient [36], as we found in this population. Regarding vitamins, the survey showed adequate values for most of the vitamins considered, as expected; however, thiamin, riboflavin, and vitamin D did not meet the recommended values. Although these data were derived from a national survey, not from a healthy control group; low intakes of calcium, potassium, iron, and vitamin D were previously reported in other diseases [37], not strictly related to CD and in different surveys [20], suggesting that much greater attention is needed.

Our study has some limitations. First, the tool adopted for estimating dietary intake, such as the 3-day food record, always needs to be interpreted with caution since individuals may consciously or unconsciously underestimate or overestimate their nutrient intake. However, the correct use and interpretation of food record might be helpful in the formulation of dietary recommendations. Second, the use of the CDAI might not be the best choice for defining disease activity, although conventionally it is accepted. However, it is extremely important to assess dietary intake according to disease activity in these patients to plan targeted dietetic approaches. Last, but not least, a matched control group could have provided further considerations about nutritional deficits in this specific population.

In conclusion, this cross-sectional study showed that the assessment of dietary intake can be crucial for identifying nutritional deficits and optimizing dietary diversity in CD patients, especially in those who are clinically quiescent. Further studies are needed to evaluate nutrient intakes in different phases of the disease, to plan focused nutrition counseling.

## Abbreviations

AR: Average requirement
BMI: Body mass index
CD: Crohn’s disease
CDAI: Crohn’s disease activity index
EI: Energy intake
DRVs: Dietary reference values
LARN: Livelli di Assunzione di Riferimento di Nutrienti ed energia per la popolazione italiana
PRI: Population reference intake
AI: Adequate intake
SFA: Saturated fatty acid
MUFA: Monounsaturated fatty acid
PUFA: Polyunsaturated fatty acid
SD: Standard deviation
